# ‘Ultrasound is an invaluable third eye, but it can’t see everything’: a qualitative study with obstetricians in Australia

**DOI:** 10.1186/1471-2393-14-363

**Published:** 2014-10-22

**Authors:** Kristina Edvardsson, Rhonda Small, Margareta Persson, Ann Lalos, Ingrid Mogren

**Affiliations:** Department of Clinical Sciences, Obstetrics and Gynecology, Umeå University, SE 901 87 Umeå, Sweden; >Judith Lumley Centre, La Trobe University, Melbourne, Vic 3000 Australia; School of Health and Social Studies, Dalarna University, SE 791 88 Falun, Sweden

**Keywords:** Australia, Clinical experiences, Clinical management, Counselling, Obstetric ultrasound, Obstetricians, Obstetrics, Perspectives, Pregnancy complications, Qualitative study

## Abstract

**Background:**

Obstetric ultrasound has come to play a significant role in obstetrics since its introduction in clinical care. Today, most pregnant women in the developed world are exposed to obstetric ultrasound examinations, and there is no doubt that the advantages of obstetric ultrasound technique have led to improvements in pregnancy outcomes. However, at the same time, the increasing use has also raised many ethical challenges. This study aimed to explore obstetricians’ experiences of the significance of obstetric ultrasound for clinical management of complicated pregnancy and their perceptions of expectant parents’ experiences.

**Methods:**

A qualitative study was undertaken in November 2012 as part of the CROss-Country Ultrasound Study (CROCUS). Semi-structured individual interviews were held with 14 obstetricians working at two large hospitals in Victoria, Australia. Transcribed data underwent qualitative content analysis.

**Results:**

An overall theme emerged during the analyses, ‘Obstetric ultrasound - a third eye’, reflecting the significance and meaning of ultrasound in pregnancy, and the importance of the additional information that ultrasound offers clinicians managing the surveillance of a pregnant woman and her fetus. This theme was built on four categories: I:*‘Everyday-tool’ for pregnancy surveillance,* II: *Significance for managing complicated pregnancy,* III: *Differing perspectives on obstetric ultrasound,* and IV: *Counselling as a balancing act*. In summary, the obstetricians viewed obstetric ultrasound as an invaluable tool in their everyday practice. More importantly however, the findings emphasise some of the clinical dilemmas that occur due to its use: the obstetricians’ and expectant parents’ differing perspectives and expectations of obstetric ultrasound examinations, the challenges of uncertain ultrasound findings, and how this information was conveyed and balanced by obstetricians in counselling expectant parents.

**Conclusions:**

This study highlights a range of previously rarely acknowledged clinical dilemmas that obstetricians face in relation to the use of obstetric ultrasound. Despite being a tool of considerable significance in the surveillance of pregnancy, there are limitations and uncertainties that arise with its use that make counselling expectant parents challenging. Research is needed which further investigates the effects and experiences of the continuing worldwide rapid technical advances in surveillance of pregnancies.

## Background

Obstetric ultrasound has come to play a significant role in obstetrics since its introduction in clinical care [1]. For some time now most pregnant women in the developed world have been exposed to ultrasound examinations in pregnancy, even though the timing of the examination and the number of scans vary considerably between countries and settings [2]. The use of obstetric ultrasound is also rapidly increasing in developing countries, particularly in urban areas [3, 4]. Ultrasound has broad application in obstetrics, including screening, diagnostics, and fetal surveillance during the course of pregnancy. The examinations are generally conducted by obstetricians or generalist physicians, radiologists, sonographers [3], or specially trained midwives [5].

The benefits of routine (screening) ultrasound include gestational age assessment, detection of multiple births, placenta localisation, assessment of fetal wellbeing, and detection of fetal anomalies [6]. Further, ultrasound plays an important role in surveillance and management of high-risk pregnancies, where its use has been shown to reduce obstetric interventions and also the risk of perinatal deaths [7]. There is no doubt that the advantages of obstetric ultrasound technique have led to improvements in pregnancy outcomes. At the same time however, it has been argued that continuing medico-technical progress has led to an increased medicalisation of pregnancy [8]. Its use has also raised many ethical challenges, especially in relation to non-medical provision [9], and its role in the practice of sex-selective abortions [10, 11], and fetal reduction in multiple pregnancies [12].

Ultrasound is generally very appealing to expectant parents [2, 13]. Expectant parents’ experiences of ultrasound examinations have been described as a confirmation of new life, meeting and connecting with the baby, and as an important step towards parenthood [14, 15]. Expectant parents in general expect a confirmation that the ‘baby’ is well and that the pregnancy is real [2, 16]. However, unpreparedness for adverse findings has been reported as common, as well as lack of knowledge about the purpose of the ultrasound examination and the limitations of the procedure [2, 13, 17]. While different aspects of expectant parents’ experiences of the use of ultrasound in pregnancy have been explored previously, there is still a gap in the literature with regards to obstetricians’ views and experiences in relation to the use of ultrasound.

We report findings from the initial stage of the CROss-Country Ultrasound Study (CROCUS) which aims to explore issues related to the use of obstetric ultrasound in both low-income and high-income countries. The specific aim of the present study was to explore obstetricians’ experiences of the significance of obstetric ultrasound for clinical management of complicated pregnancy and their perceptions of expectant parents’ experiences.

## Methods

### Outline of the CROCUS study

The CROCUS-study is a two-phase project, with qualitative and quantitative components, being undertaken in a number of low-income and high-income countries in Europe, Africa, Asia, and Oceania. This study was the first exploratory sub-study of the qualitative phase. The countries involved in the CROCUS-study have been selected to represent a variety of contexts, including culture, religion, gender perspectives, legislation, organisation of obstetric and maternal health care, and organisation of and access to ultrasound examinations during pregnancy.

### The local study setting

The Australian health care system consists of both public and private funders and providers. Medicare, the compulsory tax-funded national health insurance scheme, offers patients free public hospital treatment and access to subsidized medical services and pharmaceuticals. Voluntary private health insurance assists people with access to hospital treatment as private patient and with access to allied health services and dental services. Private medical practitioners provide most community-based medical treatment, and general practitioners (GPs) are normally the first point of medical contact in the health care system [18, 19]. Pregnant women in Australia have a range of choices for model of health care during pregnancy, birth and the postpartum period [20]. All pregnant women are offered dating ultrasound examinations, although the screening approach varies across the country [21]. In the state of Victoria, 98% have at least one ultrasound examination, with 94% having the routine anomaly ultrasound scan [22]. Women may undergo a number of ultrasound examinations during the course of pregnancy if pregnancy-related complications occur. According to the researchers’ knowledge, ultrasound examinations are in Australia predominantly performed by obstetricians or sonographers.

### Participant recruitment process

Participants for this study were recruited from two large hospitals in Victoria, Australia, each hospital with over 4000 births per year. Approval to involve obstetricians was sought from the heads of each department of obstetrics and gynecology. Inclusion criteria for participation were being an obstetrician working with obstetric ultrasound examinations as a major work task, or doing obstetric ultrasound examinations as part of their general obstetric care, or using the results of obstetric ultrasound in clinical management of pregnant women. Names and contact details of obstetricians were obtained via the department heads, who also mediated bookings of appointments for interviews. All participants were provided written information about the study aim and procedures. A convenience sample of 14 obstetricians meeting the inclusion criteria was assembled and no one approached declined participation in the study. Verbal and written informed consent was obtained prior to the start of each interview.

### Participant characteristics

Fourteen obstetricians agreed to participate; ten females and four males. Their ages ranged between 33 and 59 years (mean 43.7 years) and their experience as an obstetrician varied between 4 and 30 years (mean 15.5 years). A few of the participants were of non-Australian origin and had previously practised obstetrics overseas. All were qualified in performing obstetric ultrasound examinations.

### Data collection procedures

All interviews took place in November 2012 and were held at the hospitals during, or in close connection with, the obstetricians’ normal work shifts. An interview guide, developed by the research group and linked to the overall aims of the CROCUS study, was used to guide the interviews. The following key domains were included (topics addressed in this paper are shown in italic).

The obstetricians’ views/experiences of:

-  *The importance/value of obstetric ultrasound for clinical management of complicated pregnancy.*
-  Clinical situations where the interests of maternal and fetal health have been in conflict.
-  Whether the woman may be considered to act as an instrument for fetal treatment.
-  *The importance of obstetrical ultrasound in comparison to other surveillance methods during complicated pregnancy.*
-  If/when the fetus can be regarded as a person.
-  Situations where the fetus has been regarded a patient with his/her own interests.
-  *Their professional role in relation to other occupational groups working with obstetric ultrasound examinations or the outcomes of these examinations.*
-  *Other issues in relation ethical aspects of the use of obstetric ultrasound.*

The individual interviews were performed by two of the authors (IM and MP). All participants were asked to complete a short anonymous questionnaire with demographic questions including sex, age, qualifications and professional experience of obstetrics and obstetric ultrasound. The interviews lasted between 22 and 65 minutes (mean 37 minutes) and were all digitally recorded. After performing 14 interviews, the whole research group met to discuss whether further data collection was needed. The authors concluded that further interviews were unlikely to provide any new information and that saturation of data had been reached [23]. The interview discussions were broad-ranging and it is not possible to report all findings here. We describe those findings of central relevance to our stated aim. Remaining findings will be reported in forthcoming papers.

### Data analysis

Data were analysed using qualitative content analysis [24]. First, three members of the research group read all interviews to get a sense of the whole (KE, RS and IM). The researchers then discussed general impressions and emerging content areas. Data addressing the aim of this study were then coded by KE and selected parts were also coded by RS and IM. KE compared the codes for similarities and differences, grouped them into content areas and subsequently into preliminary categories and sub-categories. These were then reviewed by RS and IM, and uncertainties in interpretations were thoroughly discussed between the three researchers until consensus was obtained. An overall theme emerged during these discussions. All five researchers then reviewed the categories and theme against the original transcripts, which resulted in minor adjustments to the labelling and the order of categories.

### The researchers’ backgrounds

The research group represents various professional disciplines and research traditions including obstetrics and gynecology, midwifery, nursing, behavioral science, maternity services and maternal health research, public health, epidemiology, and qualitative methods. The authors’ diverse experiences added complementary perspectives in interpretation of data, something we believe enriches the overall trustworthiness of the study.

### Ethical considerations

All participation was voluntary and based on informed consent. Ethics approval was obtained from the Faculty Human Ethics Committee at La Trobe University in Melbourne (reference FHEC12/135) and the Human Ethics Committees of the two participating hospitals. To ensure confidentiality, characteristics of participants are presented only with means and ranges and the participants are referred to with individual numbers where quotations are presented.

## Results

### Obstetric ultrasound – a third eye

An overall theme emerged during the analyses, which has been termed ‘Obstetric ultrasound - a third eye’, reflecting the significance and meaning the participants placed on ultrasound in pregnancy, and the importance of the additional information ultrasound offers a clinician caring for a pregnant woman and her fetus. Yet, despite being an invaluable tool, at times this additional ‘eye’ also created situations that were difficult to deal with for both obstetricians and the women they cared for. The overall theme was built on four categories: I:*‘Everyday-tool’ for pregnancy surveillance,* II: *Significance for managing complicated pregnancy,* III: *Differing perspectives on obstetric ultrasound, and* IV: *Counselling as a balancing act*. An overview of the theme, categories and sub-categories is presented in Table 1.

**Table 1:** **Theme, categories and sub-categories**

Theme	Category	Sub-category
**Obstetric ultrasound – a third eye**	**I. ‘Everyday-tool’ for pregnancy surveillance**	An indispensable tool in current practice
		Liberal use
	**II. Significance for managing complicated pregnancy**	Highly valued surveillance method
		Essential tool in optimising pregnancy outcomes
	**III. Differing perspectives on obstetric ultrasound**	Obstetrician versus public confidence in ultrasound
		Differing expectations during examinations
		Body weight and imaging ability
		Cultural variations in expectations
	**IV. Counselling as a balancing act**	Communicating uncertain findings
		Ultrasound’s potential for harm
		Guiding through uncertainty - a rewarding mission

*‘I say to the juniors that ultrasound is your third eye and it should inform your practice.’* (Participant no 8)

### I. Everyday-tool for pregnancy surveillance

#### An indispensable tool in current practice

The participants described obstetric ultrasound as an essential and useful tool for screening, diagnosis and surveillance of maternal and fetal health. It was depicted as an integral and essential part of everyday obstetric care, in clinical management to optimise health outcomes for pregnant women and their fetuses. In addition, obstetric ultrasound was viewed as an accessible and safe screening tool, said to play a key role in identification of first trimester fetal malformations and Down syndrome screening. The possibility of screening for chromosomal abnormalities was perceived as having changed obstetric practice.

*‘I think it will remain because it’s cheap, easy and as far as we’re aware, safe. It will remain a very good screening tool.’* (Participant no 3)

*‘So we … you know by diagnosing fetal anomalies, we enable termination and clearly that’s got a role… And you know we minimise families suffering for many years looking after disabled babies.’* (Participant no 2)

Obstetric ultrasound was also described as essential for identifying the number of fetuses present and the localisation of the placenta so that the management of the pregnancy could be adjusted accordingly. Screening for other conditions, such as intrauterine growth retardation, markers for development of pre-eclampsia and fetal blood flow abnormalities were also described as highly significant for clinical management during the course of pregnancy. Overall, participants described ultrasound as an ‘everyday-tool’ they could not imagine doing without. Since its clinical introduction, ultrasound has dramatically changed the way obstetrics is practised:

*‘I think it’s been a big game changer in obstetric care and modern obstetrics is ingrained with ultrasound.’ (Participant no 10)*

Reliance on ultrasound was sometimes viewed as outweighing clinical experience. A few suggested that some clinical skills had been lost because of the increased use of obstetric ultrasound.

*‘I think it’s a very useful tool, I think we’re getting to the situation where many people can do nothing without an ultrasound, so those clinical skills have gone to a large extent and I think that’s happening in other areas of medicine, as well.’* (Participant no 3)

#### Liberal use

Obstetric ultrasound was described as being used liberally and not always with appropriate medical indications. Participants discussed the implications of too liberal use, and highlighted the risk of picking things up that they had to act upon, although in the end, findings might be non-significant. This was also viewed as contributing to unnecessary anxiety for the parents to be.

*‘The disadvantage is sometimes picking things up that you probably rather do not want to know.’* (Participant no 5)

Liberal use of obstetric ultrasound was described as occurring because of reasons such as not to be blamed by colleagues or expectant parents if major problems were not detected or anything subsequently went wrong. This was particularly mentioned in relation to more risk-averse private practitioners and junior or less experienced doctors.

*‘Most young obstetricians wouldn’t dare conduct a ward run with a consultant like me without having an ultrasound report to show a consultant, irrespective of whether the patient truly needed the ultrasound or not.’* (Participant no 11)

The obstetricians sometimes also reported performing extra scans, in some cases at every visit and without medical indication, with the main purpose of providing reassurance, particularly for women with previous adverse outcomes such as stillbirths.

*‘I see a lot of patients who’ve had previous stillbirths. For those patients I tend to do scans every time I see them because they like the reassurance.’* (Participant no 12)

The increasing popularity of non-medical, commercially driven ultrasound examinations was also mentioned as concerning, with the potential for such examinations to create unnecessary anxiety.

*‘And the advent of so called 3D and 4D ultrasound has created a new phenomenon in the community … I think the people have just learned to use the machine but they’re not even ultrasound technologists. They’ve just … they’ve learnt to use the machine to try and produce some pretty pictures, it’s entirely voluntary thing, it’s just for money. And I try to dissuade patients from doing those because I think that there’s a potential there for them to just create more anxiety.’* (Participant no 12)

### II. Significance for managing complicated pregnancy

#### Highly valued surveillance method

Obstetric ultrasound was described as an outstanding surveillance method compared to other methods used during pregnancy. The interviewed obstetricians were unanimous that ultrasound was the best means of monitoring the fetus during pregnancy prior to labour, as it provided more information than for example a CTG (cardiotocogram) about a fetus’ wellbeing.

*‘I think ultrasound is really only … your only good way to [conduct] surveillance [of] the baby.* […] *I don’t see any other really … once you know that there is something, you need the ultrasound to kind of, yeah, get you further.’* (Participant no 5)

#### Essential tool in optimising pregnancy outcomes

Obstetric ultrasound was seen by all as an essential tool for decision-making; a tool that they could not do without in the management of complicated pregnancy. Participants described its central role in optimising pregnancy outcomes, whether for intervention or non-intervention, in different clinical situations. For example, the use of obstetric ultrasound could allow obstetricians to plan the timing of the delivery more optimally.

*‘Making sure that we try to get the timing of delivery right, not too early and not too late. And that’s really the most powerful application of ultrasound I think.’* (Participant no 1)

The use of obstetric ultrasound was also described as contributing to clinical preparedness for fetal problems. Detecting problems such as heart abnormalities meant that women could deliver at the appropriate sites and the health care of the fetus could be planned in advance. The obstetricians all believed that the management of complicated pregnancy had improved significantly over the years due to the advances made in obstetric ultrasound. There was universal agreement on the positives of early detection and diagnosis of clinical problems via obstetric ultrasound, with improved survival rates and prognosis for many fetal conditions.

*‘I think that’s very important, we know that at least 3% of the babies they have some sort of malformation and that the majority of them can be diagnosed by ultrasounds. Also we have some maternal complications such as pre-eclampsia, for example, it’s the most common one, or placental insufficiency that’s causing intrauterine growth restriction that we can do the diagnosis of during the pregnancy… these kind of risks and you can try to prevent. I think that ultrasound is so important for this management nowadays.’* (Participant no 4)

### III. Differing perspectives on obstetric ultrasound

#### Obstetricians versus public confidence in ultrasound

Obstetricians reported that in general, expectant parents put a very high value on obstetric ultrasound examinations; expectant parents had high expectations, saw its role as important and had a high level of trust in the examination. They had also experienced a general increased interest in obstetric ultrasound in the community. Expectant parents’ desire for additional scans was commonly mentioned and the obstetricians sometimes commented on the disappointment expressed by women if they were not offered scans during visits. Reassurance that everything was fine was described as the main expectation of the examination by expectant parents, and also the incentive for wanting additional scans. The obstetricians saw expectant parents as believing that the obstetric ultrasound examination would provide a clear-cut answer whether something was wrong or not.

*‘Lots of parents I think do their 20 week scans because they want to be reassured that everything is fine.’* (Participant no 5)

Obstetricians’ narratives also reflected that these days there were few safety concerns around ultrasound among expectant parents. Rather, they frequently mentioned that expectant parents did not always understand the limitations and potential disadvantages of pregnancy ultrasounds, such as false positive and false negative findings.

*‘I think… most members of the public think an ultrasound is a more powerful tool than it is.’* (Participant no 11)

Some also felt that even their midwife colleagues sometimes had a somewhat naive understanding of obstetric ultrasound with regard to the consequences of false positive and false negative findings.

*‘I think from my observation of most midwives, they’re probably a little bit more inclined to be like members of the public, I think they place too much emphasis on what an ultrasound can do and tell you.’* (Participant no 11)

Some of the obstetricians perceived expectant parents’ confidence in obstetric ultrasound as somewhat misguided, and presented the view that ultrasound nowadays has become so good that parents expect it to provide accurate answers in all instances. To avoid such situations, obstetricians frequently described how they tried to inform pregnant women about the limitations of the examination.

*‘Now there’s the expectation that if the baby is born with a fetal malformation, the first question’s asked: Well why wasn’t it picked up on ultrasound?’* (Participant no 14)

*‘Patients think that having had the 20 week morphology scan then all horrible abnormalities must be excluded, but we all know from experience that there are brain malformations for example [that] don’t really appear until more like the beginning of the third trimester.’* (Participant no 12)

During the interviews, obstetricians pointed out that no test is perfect and that mistakes and uncertain findings could occur at any point during the pregnancy. Despite being aware of these limitations, there was indeed disappointment even for the obstetricians when a deviation was not picked up by ultrasound.

*‘I think as much as the patients have high expectations of what medical care and interventions do, so do we.’* (Participant no 7)

*‘That would-be down side of the area of medicine we’re in is that the tension or the pressure is on, not to miss anything. That has increased dramatically.’* (Participant no 14)

#### Differing expectations during examinations

A shared experience among the obstetricians was that there was a mismatch between expectant parents’ expectations of obstetric ultrasound examinations and their own medical perspectives. They commonly described how pregnant women and their partners were focused on getting good images of the fetus during the examination, how they asked for photos and wanted to find out the sex of the fetus, although this was not the aim of the scan from a medical perspective. Obstetricians frequently described situations where the expectations differed, and the most distinct was when they were asked for ‘pretty pictures’ during an examination while their own focus was on identifying abnormalities. The obstetricians felt that expectant parents may not always fully understand the aim of, or the indication for the examination.

*‘It’s not uncommon that people come in and say oh, can you take a photo of the baby and also finding out the sex of the baby even though it’s not the point of the scan at all, but you know, it’s interesting that the parents' expectations of what the scan is all about differ quite a lot from your expectations I think.’* (Participant no 6)

The pregnant women and their partners were seen as sometimes conceptualising the examination as an event and an experience to share with family and friends. This was viewed as further indication of some parents’ lack of awareness of the real purpose of the examination, and subsequently a lack of preparedness for any adverse finding. Although described as infrequent, obstetricians perceived these situations as challenging if the examination detected any significant abnormality.

*‘That’s always a delicate situation when you’re faced with fetal malformation and you have four or five people in the room, as to how you best manage it.’* (Participant no 14)

#### Body weight and imaging ability

The dilemmas posed by obesity and increasing weight in pregnant women were raised in relation to ultrasound performing less well as regards imaging quality in women overweight or obese. At the same time, obstetricians commented that these factors also put women at higher risk for abnormalities, but pointed out that overweight and obese women’s expectations of obstetric ultrasound examinations were in general as high as those of their normal weight peers.

*‘One of our problems with ultrasound is increasing obesity. So our imaging ability on those women is not as good and their expectations are not less.’* (Participant no 2)

The obstetricians believed that many women were unaware of what problems overweight and obesity could confer in the context of obstetric ultrasound examinations. Some even thought that increased weight in the pregnancy population outweighed the advances made in ultrasound imaging resolution. Paradoxically, the increasing problems with overweight and obesity also resulted in obstetricians performing more obstetric ultrasound examinations because of the difficulty of assessing the pregnancy clinically.

*‘These women are more at risk of abnormalities, so obesity increases your risk and makes them harder to diagnose as well, so I think it really truly is a growing problem.’* (Participant no 3)

*‘They really don’t seem to understand that the technology is such that the fatter you are the less you’ll see.’* (Participant no 3)

#### Cultural variations in expectations

The obstetricians pointed out that the childbearing population in Australia comprises many different nationalities and backgrounds, and expectations about ultrasound were described as being in part culturally dependent. The obstetricians commented that women often expected what is common practice in their home countries, which meant that some pregnant women expected ultrasound to be performed at every appointment while others thought that obstetricians were doing too many scans.

*‘Italian patients think that they ought to have ultrasound every time they see the obstetrician and they feel short changed in some form that you’re doing less than what you’re meant to do if there’s no ultrasound. On the other hand, if you have a Scandinavian patient or a British patient they might think that you’re doing too much or doing unnecessary things if you propose that you want to scan them at each visit, so there is that cultural difference.’* (Participant no 8)

### IV. Counselling as a balancing act

#### Communicating uncertain findings

Clinical situations characterised by uncertainty were frequently mentioned during interviews as something the obstetricians struggled with, especially in relation to counselling expectant parents. The most challenging situations described were those where the scan provided signs of some pregnancy deviation, but where the obstetrician did not know the significance of the finding or even if it could be considered abnormal. Communicating such findings to expectant parents was unanimously described as difficult.

*But there are of course sometimes those minor things that you can see that makes you feel uncomfortable, but you can’t … yeah, and you explain it to patients but then you have the whole uncertainty building up and you can’t always have a clear-cut answer. And I think that is hard. That is the hardest bit.’* (Participant no 5)

*‘I think the problem starts by picking things up, and some things are very clear… but some things aren’t that clear and then you come into that ethical dilemma of what you tell people and how far you’re going to take things.’* (Participant no 5)

Uncertain answers arising from difficult to interpret ultrasound findings were described as hard to deal with for the expectant parents, and some participants thought that certain answers were easier to manage, even when problems were identified.

*‘If one is certain about what’s going on, it’s … it’s easier for patients to accept, almost no matter how … no matter how bad it is.’* (Participant no 1)

*‘You know your baby has this, it has a 20% chance of being very severely disabled, a 30% chance of mild disability, a 50% chance of being normal. And for many families that’s unmanageable and I can see how that would be unmanageable.’* (Participant no 2)

Sometimes a second or third opinion was seen as helpful and important in situations of uncertainty. Meeting an additional expert could also allow expectant parents more opportunity to absorb the information. The obstetricians emphasised the importance of phrasing their sentences in the right way when delivering uncertain answers, as they were conscious that how they conveyed information would have a significant influence on expectant parents’ decisions. Examples were given of women who wanted to deliver prematurely, or asking for a termination, although the obstetricians were not certain that the prognosis for the fetus was worrying.

*‘But some people can’t live with that uncertainty and they request terminations of pregnancy, which then is hard because we kind of don’t know what the actual prognosis would be.’* (Participant no 5)

The obstetricians found it particularly difficult to get complex information across to people with lower levels of education, or where there were language barriers and counselling occurred via interpreters.

*‘It is sometimes very hard for us even to comprehend what’s going on, and then to kind of get that information across to a patient. And especially when they come from a lower socioeconomic area or aren’t … you know, sometimes you really need to be quite educated to understand what we’re all saying.’* (Participant no 5)

Obstetricians experienced it as essential to be able to discuss findings with the pregnant woman immediately after a scan, and to answer questions immediately. Some thought that this was a problem when other occupational groups performed the scans (sonographers), as expectant parents could believe that something was wrong if their questions could not be answered straight away.

*‘The patients have the benefit of seeing what we’re pointing out and then we get the discussion straight away. I think it’s important for us to be able to counsel based on our own findings and also have an immediate answer to a problem, so that when we see a problem then we can discuss it straight away.’* (Participant no 7)

#### Ultrasound’s potential for harm

Obstetricians acknowledged that there were also situations where the use of ultrasound had caused more harm than good, as deviations picked up that were not clear could create a lot of anxiety and stress. Many examples were provided of false positive findings that had caused distress or even ruined the experience of the pregnancy. Participants clearly indicated that there were expectant parents for whom the pregnancy was made more stressful by the use of ultrasound.

*‘The more you see sometimes the more uncertain things get. And you can ruin a pregnancy quite a bit like that. So I’m not sure whether it’s always good.’* (Participant no 5)

*‘So we’ve all caused harm, certainly significant psychological harm by the things that we’ve done with ultrasound.’* (Participant no 2)

The participating obstetricians noted that after receiving information about possible abnormalities, expectant parents could often not relax until the baby was born and someone reassured them that everything was fine. Further, some obstetricians believed that the worry and anxiety caused by the ultrasound examination could have long lasting effects; they believed that the anxiety about something being wrong with the child sometimes lasted throughout the child’s upbringing.

***‘****I don’t think they ever get over that concern. And then even worse, the ones where we just are unsure all the way through. And then I … you know we don’t always see them in the long term but I can imagine that those women … when the baby’s one and two and three, they’re still not really quite … that that concerns me.’* (Participant no 2)

*‘Those people are angry and bitter later when the child is normal and the pregnancy’s been overshadowed by a lot of distress because of comments at the time of the ultrasound but it … a lot of stuff is grey and it’s hard to get it right but there are definitely patients for whom we make the pregnancy more stressful.’* (Participant no 9)

#### Guiding through uncertainty - a rewarding mission

Although dealing with difficult decisions in their everyday practice, the obstetricians all described high levels of satisfaction in their work. They felt that they were in the position to make a significant difference to people’s lives, and achieving the best possible outcomes in spite of complexities was described as providing great satisfaction. They also believed that their skills and judgement were highly valued by expectant parents.

*‘I know that I am providing good care to women at a terrible time in their lives. And whether the outcome is good or bad, they know that what could have been done, reasonably was done, that it was done by people who cared about them and knew what they were talking about. And that’s very rewarding.’* (Participant no 2)

Counselling expectant parents was unanimously described as a particularly rewarding aspect of their work. Following a woman through complicated pregnancy was portrayed as a journey, and although also described as difficult at times, the obstetricians generally found this journey of counselling very fulfilling. This was so irrespective of whether the pregnancy outcomes were good or bad. In fact, the interviewees provided many examples where they felt that their efforts had made an important difference, even though the pregnancy outcome had been poor.

*‘I think that’s most rewarding, just guiding them through all those decisions which again I think are very hard decisions for parents to make.’* (Participant no 5)

*‘I enjoy the counselling side of it and yeah, the difficulties of not having a right or wrong answer and try to, you know, help couples through what is probably one of the most difficult decisions in their life and making it a little bit easier if I can, and for them having happy memories of their management and treatment through what was a difficult time.’* (Participant no 7)

The obstetricians described teamwork with other doctors, midwives, psychologists or other professionals as essential in relation to counselling and providing support to expectant parents in complex situations. They also felt very strong support from obstetrician colleagues as they could exchange those experiences that are exclusive to obstetric practice.

*‘So you know we’re not in this alone… the shared experience … that’s the good thing about working in a larger centre, we’ve all been through it all.’* (Participant no 1)

Being able to reassure expectant parents that everything seemed okay, particularly in cases where fetal abnormalities had been suspected, was also described as one of the most rewarding moments.

*‘The best part of my work? I think it’s reassuring parents, because I see lots of referrals that turn out not to be what people thought. It’s to be in a position of scientific knowledge and be able to tell parents no, this is not exactly as someone else thought. It’s going to be alright.’* (Participant no 10)

## Discussion

The aim of this study was to explore obstetricians’ experiences of the significance of obstetric ultrasound for clinical management of complicated pregnancy and also their perceptions of expectant parents’ experiences. Although the aim was to focus primarily on complicated pregnancy, participants raised dilemmas in relation to all aspects of use of ultrasound.

Results from the present study show that obstetric ultrasound was an essential tool (‘third eye’) for obstetricians in surveillance of maternal and fetal health. More importantly however, the study highlights some issues and dilemmas that arise with its use; obstetricians’ and expectant parents’ differing perspectives and expectations of obstetric ultrasound examinations and the challenges related to uncertain findings, and how the obstetricians try to balance this in their counselling and discussions with expectant parents prior to, during, and following an ultrasound examination. This is to our knowledge the first study to explore these experiences and views among obstetricians, contributing new knowledge to the field.

It is clear from this study that ultrasound is a highly valued tool for obstetricians in their everyday practice and for managing complicated pregnancy. However, they experienced public confidence in obstetric ultrasound to be somewhat misguided and saw a gap between their own and expectant parents’ knowledge about the possibilities and drawbacks of the use of obstetric ultrasound. These findings seem consistent with those from previous studies. For example, a systematic review of women’s views of pregnancy ultrasound in 18 countries including Australia, revealed that women are often unprepared for adverse findings, and often lack information about the purposes of the examination and about the technical limitations of the procedure [2]. Several studies have also shown that women’s understanding of ultrasound often does not meet the requirements of informed choice [2, 25–27], which includes the three components information, comprehension and voluntary choice [28]. While women may be familiar with the practical aspects of prenatal examinations, their understanding of the drawbacks and other consequences of the tests are often insufficient [26]. Probably, these issues are more related to routine antenatal ultrasound use (screening) than the use of ultrasound in the management of complicated pregnancy. The present study has illustrated how the obstetricians experienced and tried to deal with challenging circumstances when pregnant women may lack understanding or have unrealistic expectations.

Results of this study also illuminate the difficulties obstetricians can encounter in clinical situations characterised by uncertainty. Communicating uncertainty was depicted as one of the most demanding aspects of obstetric practice. During routine pregnancy ultrasounds, expectant parents in general expect reassurance that everything is fine [2, 16]. The obstetricians are obliged to provide objective information, however, as indicated by our participants, this information may be difficult for them to formulate appropriately and hard for the expectant parents to interpret and put into perspective. This may create an unbearable situation for some women and men, characterised by anxiety and worry for the wellbeing of the fetus/the future child. While previous studies investigating obstetricians’ experiences of communicating uncertainty following ultrasound examinations are lacking, communicating uncertainty and assessing expectant parents’ understanding have been pointed out as among the most challenging aspects in other areas of obstetric care [29]. Obstetricians are expected, as are doctors in general, to cope with the complexity of diagnoses and decisions, while simultaneously being sensitive to the feelings of the pregnant woman and her partner when communicating uncertainty or breaking bad news. This is a demanding task in which the obstetrician also needs to be in possession of emotional competence and good communication skills. The pregnant woman deserves to be managed by an astute and empathetic obstetrician and a skilled team. As some diagnoses may cause a psychological crisis, there is often need for psychological and social support in parallel with the obstetric investigation and treatment, as the obstetricians also pointed out. When communicating uncertainty or breaking bad news, the obstetrician and the rest of the team have a responsibility to help the couple to put the problem into perspective and navigate their way forward. Following expectant parents through this journey was depicted by the participants as a difficult task, but at the same time as one of the most rewarding aspects of being an obstetrician. Despite both doctors and patients likely agreeing on the importance of information provision and support, there can however, be discordance in the approach health care providers have, and the approach preferred by pregnant women and their partners [30]. For example, while providing ‘hope’ and supplementary information were emphasised as important to expectant parents who were at risk of giving birth to an extremely premature infant, health care providers expressed the importance of ‘objectivity’ and emphasised the desire not to provide ‘false hope’ [30].

The obstetricians in the present study believed that the increased stress and anxiety as a result of not knowing the significance of abnormal findings affected the pregnancy experience very negatively for some women. These findings are congruent with findings from previous studies in pregnant women that have shown that unpreparedness for adverse findings can make this information traumatic [2, 31, 32]. Interestingly, in a Swedish interview study, it was found that the majority of women who had soft markers detected via routine ultrasound would rather not have known, or were hesitant about getting this information [17]. On the contrary, in a prospective Canadian survey of women booked for a (routine) anatomy ultrasound examination in second trimester, only 6% of women were hesitant or clearly did not want to know about soft markers if they were seen. However, 23% of the study participants stated that soft markers should be reported only after the woman has been counselled and given her consent [33]. Our study, which is the first to report obstetricians’ views on the use of ultrasound, in conjunction with the number of publications that report on women’s limited understanding of the purpose and potential of the scan [2, 13, 17], provides evidence for the utter importance of appropriate counselling prior to the examination. Thus, improved information about the ultrasound examination, including possibilities and limitations, seems to be the most appropriate way for obstetricians and other caregivers to minimise the psychological impact of uncertainty, as well as to establish informed consent to the scan. This is becoming increasingly important considering the effects of the rapid technical advances, the growing diagnostic possibilities, and the more widespread use of ultrasound.

Our findings also highlight the obstetricians’ perception of their own and their patients’ perspectives and expectations of obstetric ultrasound examinations, and how these at times conflicted. Most evident was the clash between the obstetricians’ medical approach to ultrasound and their opinion that some expectant parents viewed pregnancy ultrasound as not only a medical procedure, but also a social event or even as entertainment. This finding is consistent with previous research [17, 34]. Taylor (2008) [35] describes the ultrasound examination as a ‘hybrid practice’, by having nonmedical meanings and functions commonly incorporated into medical ultrasound practice. This ‘hybrid practice’ is exemplified through the way ultrasound equipment is often designed to facilitate the ‘entertainment’ aspect through swivel monitors to enable expectant parents to follow the examination, and special printers available for providing keepsake photos [35]. The growing phenomenon of non-medical or so-called ‘entertainment’ ultrasound was raised as a concern among obstetricians. Non-medical use of obstetric ultrasound can be defined as situations where the purpose of the examination is solely to view the fetus, take its picture or determine its sex without medical indication [36]. An ethical analysis of non-medical fetal ultrasound concludes that obstetric ultrasound practice is ethically justifiable only if the indication for its use is based on medical evidence [9]. Furthermore, the practice of non-medical or ‘entertainment’ ultrasound is discouraged by governments and professional bodies [9, 37–41]. Reasons for seeking non-medical fetal ultrasound have been reported as dissatisfaction with the medical imaging, (including factors such as not learning the sex of the fetus), insufficient length of the obstetric ultrasound appointment, poor visual quality of images or unfriendly staff [34]. Other non-medical reasons for participation in ultrasound screening include to experience the pregnancy as more real and also to give the partner the opportunity to experience and see the pregnancy [42], as undergoing ultrasound provides an excellent opportunity for the expectant father to meet and connect with the fetus [14].

Findings in the present study suggest that ultrasound was sometimes used liberally in clinical practice and not always according to medical criteria for reasons such as risk-averseness, lack of clinical experience, or expectant parents’ need of reassurance. It is plausible that this is not unique to this specific study context. Although the benefits of ultrasound are well established in relation to clinical management and maternal and fetal health outcomes [6, 7], its use also carries some risks. These can broadly be categorised into diagnostic errors (e.g. overdiagnosis/underdiagnosis) and possible biological effects [43]. The obstetricians in our study repeatedly described experiences related to the former: the psychological harm caused due to false positive or false negative findings, or the stress caused when the information obtained from an examination was too difficult to grasp or manage for expectant parents. Interestingly, in this study the possible biological fetal effects were not given much attention by the participating obstetricians, the implications of too liberal use was exclusively discussed in relation to diagnostic errors. This may have several explanations, including the fact that ‘risks’ was not raised as a separate topic for discussion by the interviewers or that ultrasound may be considered safe, as some of the participants stated. That pregnancy ultrasound does not pose any risk to a pregnant woman or fetus has been suggested by others to be a common perception among clinicians [43]. Further studies from the US and Europe have shown poor knowledge among those performing obstetric ultrasound examinations regarding aspects of fetal safety during pregnancy [44, 45]. However, to our knowledge, there is no such data for Australian obstetricians and further studies into this area seem needed. There is to date not sufficient evidence about the absolute safety of pregnancy ultrasounds. Although previous systematic reviews regarding safety have been unable to find evidence of significant adverse maternal, perinatal, or childhood outcomes [6, 46], an association between exposure to ultrasound in pregnancy and non-right handedness has been found [46, 47]. More studies around safety are warranted [46, 48], especially since experimental studies on fetal mice have shown adverse effects [49], and as the most recent systematic review on safety is based predominantly on ultrasound exposures before the mid 1980s, when the acoustic potency of the ultrasound equipment was much lower than today [46], and the use of ultrasound less frequent. The uncertainties regarding safety are evident in the recommendations of prudent use and adherence to the ALARA (As Low As Reasonably Achievable) principle [41, 46].

One of the relatively novel aspects that emerged in our study was the issue of pregnant women’s body weight in relation to imaging performance. This is an escalating issue in obstetrics due to the increasing trends of overweight and obesity in childbearing women [50]. It has been shown that image quality substantially decreases with increasing BMI, which in turn negatively impacts on the detection rate of congenital anomalies [51]. This is problematic especially considering that the risk for adverse fetal and pregnancy outcomes also increases with increasing BMI [52, 53], as pointed out by the participants in our study. Previous research has shown a low level of awareness of maternal and offspring risks linked to overweight and obesity [54–57]. However, research into women’s understanding of the influence of BMI on image quality, and obstetricians’ experiences of counselling overweight or obese women in relation to pregnancy ultrasounds is lacking. A study conducted in the UK showed that the ultrasound examination was a significant source of distress in women who were obese if difficulties imaging the fetus were not clearly explained during the examination [58]. Thus, skirting around the issue because of fear of upsetting, stigmatising or blaming women [59] may be an unfortunate strategy, especially when overweight or obese women expect the same outcome of the examination as their normal weight peers, as indicated in our study. Obese pregnant women are in general sensitive about their size [58], however, caregivers’ vagueness and inconsistent messages can make women feel even more uneasy about their weight [60]. Thus, providing adequate and consistent information [60], and having an affirmative approach are ways for care providers to alleviate discomfort and increase wellbeing in obese women during consultations [61].

### Strengths and limitations

Factors increasing credibility in this study include that obstetricians with varying characteristics in relation to gender, age, length of experience in obstetric practice, and work settings (two hospitals with different characteristics) were recruited, and that none of the approached obstetricians declined participation. Furthermore, the participating obstetricians were highly engaged and reflected extensively on their work during the interviews. This contributed to a broad range of topics and perspectives being raised with us, providing a comprehensive picture of the phenomenon under study [24]. We aimed to increase dependability by consistency in data collection and analysis. The use of an interview guide ensured coverage of all topics, at the same time as it prevented interruption in the obstetricians’ narratives, as questions were not asked in a predefined order. All interviews were performed by two researchers (IM and MP) within a two week period, which further increased dependability in data collection. The authors also collaborated closely during data analysis and reporting to further strengthen the dependability and credibility of the study [24]. It is important, however, to bear in mind that the findings presented in this study are only representative of the experiences and views expressed by the participating obstetricians. Although it is likely that many of the aspects discussed are transferable to obstetricians working in other high-income settings, context-specific factors such as organisation of services, culture, gender perspectives, religion, legislation, and economy may influence experiences with pregnancy surveillance, clinical management and utilisation of obstetric ultrasound.

## Conclusions

This study highlights a range of previously rarely acknowledged clinical dilemmas that obstetricians face in relation to the use of obstetric ultrasound. Despite being a tool of considerable significance in the surveillance of pregnancy, there are limitations and uncertainties that arise with its use that make counselling expectant parents challenging. Research is needed which further investigates the effects and experiences of the continuing worldwide rapid technical advances in surveillance of pregnancies.
